# Lung health in Africa: African solutions for African challenges – to infinity and beyond

**DOI:** 10.7196/AJTCCM.2018.v24i1.210

**Published:** 2018-04-03

**Authors:** Coenie Koegelenberg, Keertan Dheda

**Affiliations:** 1 Division of Pulmonology, Department of Medicine, Stellenbosch University and Tygerberg Academic Hospital, Cape Town, South Africa; 2 Division of Pulmonology, Department of Medicine and UCT Lung Institute, University of Cape Town, South Africa

## Editorial

Africa is a place of dichotomy. Our continent is crippled by the ‘colliding epidemics’ of tuberculosis, HIV and smoking-related diseases, but it is also a place of exceptional beauty and great abundance, with a communal spirit and a considerable amount of hope. The unique challenges of our resource-limited environment require unique and innovative solutions. Africa must, and can, provide solutions to our own health crises. Our health-related research, whether it be basic science, epidemiological, or clinical, must be relevant to Africa, and must be read, analysed and implemented in Africa. It is only through this type of capacity development that we will create a multiplier effect. Therefore, there is a niche for and an unmet need to provide a high-quality pulmonary and critical-care forum on a continental level that speaks to the needs and aspirations of the African continent (and that is not currently covered by other journals, where intense competition for space has ‘crowded’ out Afrocentric views and perspectives). There are also business concerns and reviewer fatigue, such that only a few journals that are that are owned and run by academic organisations, and well supported, are likely to thrive.

This year marks the 24th year of publication of the journal of the South African Thoracic Society (SATS), and the year that it transforms from the *South African Respiratory Journal (SARJ)* to the *African Journal of Thoracic and Critical Care Medicine (AJTCCM)*. With the recent surge in submissions from authors based in other African countries, the journal has the potential to grow into the flagship thoracic journal of the African continent. Expansion is now assured, as leading academics from African countries other than South Africa (SA) are now being incorporated into the journal’s editorial board. Furthermore, this year also marks the first combined SATS - Pan African Thoracic Society congress to be held in Durban, SA in April 2018.

The journal will take further leaps forward with its likely accreditation with PubMed in early 2019. Additionally, we are accepting international submissions, which will greatly boost our circulation.

The *AJTCCM* aims to be the ideal platform for African basic scientists, epidemiologists and healthcare workers to disseminate their knowledge, research and experience. Is this an SA ‘big brother’ move to dominate and take charge? Absolutely not! Instead, we see the *AJTCCM* as a unifying initiative, and we envision that it could serve as a platform of excellence for African pulmonary science, and a dissemination platform for many stakeholders, including those from the academic, private and government sectors. Indeed, journals like *Respirology* and *Respiration* serve as proof of concept of providing a publication platform for stakeholders from many different countries.

We call on the thoracic and critical care fraternities to support our new venture by submitting manuscripts to the *AJTCCM* for publication. Let us join hands and make African excellence known to the world!

